# Investigation of the status of immune checkpoint molecules (PD-L1 and PD-1) in meningiomas by immunohistochemistry

**DOI:** 10.55730/1300-0144.5843

**Published:** 2024-04-02

**Authors:** İsmail SAYGIN, Emel ÇAKIR, Seher Nazlı KAZAZ, Ali Rıza GÜVERCİN, İlker EYÜPOĞLU, Müşerref Müge USTAOĞLU

**Affiliations:** 1 Department of Pathology, Faculty of Medicine, Karadeniz Technical University, Trabzon, Turkiye; 2 Department of Pathology, Sancaktepe Şehit Prof. Dr. İlhan Varank Training and Research Hospital, İstanbul, Turkiye; 3 Department of Oncology, Faculty of Medicine, Karadeniz Technical University, Trabzon, Turkiye; 4 Department of Neurosurgery, Faculty of Medicine, Karadeniz Technical University, Trabzon, Turkiye; 5 Department of Radiology, Faculty of Medicine, Karadeniz Technical University, Trabzon, Turkiye

**Keywords:** PD-L1, PD-1, meningioma, immunotherapy, immune checkpoint

## Abstract

**Background/aim:**

Meningiomas are the most common primary brain tumors of the central nervous system. Immunotherapy is a promising treatment method applied in many types of cancer. There is no standard and effective medical treatment to reduce recurrence and mortality in cases of incomplete resection of meningiomas and in high-grade cases. In order to investigate medical treatments in addition to surgery and radiotherapy, in this study, the status of immune checkpoint molecules (PD-L1/PD-1), which are the target of immunotherapy, in meningiomas was investigated.

**Materials and methods:**

Four hundred two cases of meningioma diagnosed between 2007 and 2020 at our institution were used. New blocks were prepared from the appropriate blocks of the cases using the tissue microarray method. Sections obtained from these blocks were immunohistochemically stained with PD-1 and PD-L1 antibodies. Obtained data were interpreted with statistical analysis.

**Results:**

Expression of PD-L1 was observed in 28.4% of meningiomas. Staining rates are higher in high-grade tumors. The staining rate of PD-L1 in the tumor increased significantly with pattern loss. PD-L1 expression in immune cells is 19.9%. Immune cell expression and the number of expressing immune cells correlate with spontaneous necrosis. Immune cell expression and the number of expressing immune cells are increased in high-grade meningiomas. PD-1 expression in immune cells is 9.0%, and this correlates with brain invasion.

**Conclusions:**

With these data, it was observed that the expression of immune checkpoint molecules PD-L1 and PD-1 increased especially in high-grade meningiomas. It may be the subject of research that these molecules may be targets of immunotherapy in the treatment of meningiomas.

## 1. Introduction

Meningiomas are the most common primary brain tumors of the central nervous system [1]. They commonly occur in middle-aged adults and exhibit a higher incidence in women than in men. Meningiomas are relatively common and typically benign intracranial tumors that in many cases can can often be managed with surgical resection. However, high-grade meningiomas, in particular, tend to grow rapidly and recur frequently even after treatment, resulting in a poor prognosis [2].

The World Health Organization (WHO) classifies meningiomas into three grades. For patients with grade 1 meningiomas, 10-year overall survival is approximately 80%–90%, and progression-free survival is 75%–90%, depending on the completeness of the resection, age of the patient, and location of the tumor [3]. Grade 2 meningiomas are characterized by the presence of 4 to 19 mitoses per 10 high-power fields (HPF), invasion into brain tissue, or meeting 3 out of 5 minor criteria (pattern loss, spontaneous necrosis, small nucleus, macronucleolus, and hypercellularity). In grade 2 meningiomas, the 10-year overall survival is 53%–79%, and progression-free survival is 23%–78% [3]. Grade 3 meningiomas are the least common group and are defined by having 20 or more mitoses in 10 HPF. In grade 3 meningiomas, the 10-year overall survival is 14%–34%, and they show rapid progression [3].

Total excision of the tumor is the most effective method in the treatment of meningiomas. However, some meningiomas show recurrence or malignant transformation even if they are totally removed. According to the Simpson Grading System, the amount of resection closely correlates with a progression-free survival [4]. Chemotherapy has no effect on meningiomas. The most important cause of recurrence is subtotal excision. Recurrence rates of up to 40% have been reported even in cases with total excision [5,6]. In cases where surgery cannot be performed and the adverse effects of radiotherapy are not desired, there is no standard and effective medical treatment for meningiomas regardless of tumor grade [7]. The absence of an effective medical treatment, especially in high-grade meningiomas that cannot be operated, negatively affects patient survival.

Recent developments in cancer treatment are especially in the field of immunotherapy targeting immune checkpoints. Immunotherapy is used in the treatment of many tumors, especially melanomas and lung tumors. New studies with immunotherapy agents continue to be investigated in different cancer types, at different stages and in combinations with other agents, and are increasingly getting ahead of other treatments.

Immune checkpoint molecules are cell membrane proteins that regulate the immune response. An immune checkpoint has two components. Molecules expressed on immune cells are generally called immune checkpoint receptors, while molecules expressed on antigen-presenting cells, tumor cells, or other cell types are called immune checkpoint ligands [8]. While some receptors stimulate the effective functioning of defense cells at immune checkpoints, some cause their inactivation with the opposite effect.

Overexpression of immune checkpoint molecules by tumor cells affects tumor-specific T-cell immunity in the cancer microenvironment. In this case, tumor cells escape from the immune system. The immune escape mechanisms of tumors using these checkpoints can be repaired with antibodies that block the inhibitory receptor-ligand interaction and thus inactivate the immune checkpoints [9].

Moreover, tumors containing tumor-infiltrating lymphocytes (TIL) and associated with a depleted phenotype often coexpress multiple immune checkpoint receptors due to chronic antigen stimulation. T cell depletion is characterized by the disruption of effector cytokine production and cytolytic function of T cells [10]. Ultimately, because tumor immune resistance and T cell depletion are characterized by coexpression of multiple immune checkpoint pathways, it may require a combined immunotherapy approach to establish successful immunocheckpoint blockade and a better antitumor immune response.

Studies show the presence of immune cells in almost all meningiomas. It is known that meningiomas are infiltrated by immune cells, including microglia, macrophages, B cells, and T cells. Macrophages are the most common immune cells in the meningioma microenvironment. Macrophage counts are higher in grade 2 and 3 meningiomas compared to grade 1 [11]. TILs in meningiomas are mainly T cells. CD8+ cytotoxic and CD4+ helper T lymphocytes are the second most common immune cells; these are followed by regulatory T cells (Treg) and natural killer (NK) cells [11].

All these data suggest that there is an immune microenvironment that may influence tumor progression in meningiomas. The immune microenvironment of meningiomas is a complex area of immunomodulatory protein expression, genetic alterations, and tumor-immune cell interactions. Therefore, we aim to contribute to the development of new personalized treatment strategies by investigating the status of immune checkpoint molecules in meningiomas.

## 2. Materials and methods

### 2.1. Samples, clinical and radiological data

In this retrospective study, 402 meningioma cases diagnosed between 2007 and 2020 in our laboratory were used. Consultation cases and cases with insufficient tumor tissue were excluded. In 315 of the cases, the patients underwent complete resection and 45 patients underwent partial resection. All cases were included after reevaluation by two neuropathologists and after confirming their morphological findings. The clinical and radiological data of the patients were obtained through the Data Processing Center of our hospital.

### 2.2. Preparing paraffin blocks

Using the appropriate paraffin blocks of the patients, new blocks containing multiple tissues were prepared with the tissue microarray method. For this purpose, tissues with a diameter of 3 mm were removed from each block with a skin biopsy apparatus, and new blocks were prepared, each containing tumor tissue from 9 patients. Sections were taken from these blocks for immunohistochemical staining.

### 2.3. Immunohistochemical antibody and staining

Immunohistochemical staining with PD-1 and PD-L1 antibodies was performed with Ventana’s BenchMark Ultra automatic staining device. The antibodies used were PD-L1 (Ventana SP263 clone) ready-to-use antibody, PD-1 (Cell Marque NAT105 clone) ready-to-use antibody. Slides stained with antibodies were evaluated by two neuropathologists under an Olympus BX51 light microscope.

### 2.4. Immunohistochemical evaluation

In immunohistochemical examination, cytoplasmic/membranous staining of PD-L1 in meningeal cells was evaluated. For PD-L1, the staining pattern and percentage in tumor cells were performed as suggested by Fehrenbacher et al. (immunohistochemical staining <1% = score 0; ≥1% and <5% = score 1; ≥5% and <50% = score 2; ≥ 50% = score 3) [12]. The staining rate of PD-L1 and PD-1 in tumor-infiltrating immune cells were also assessed as suggested by Fehrenbacher et al. (immunohistochemical staining <1% = score 0; ≥1% and <5% = score 1; ≥5% and <10% = score 2; ≥ 10% = score 3) [12]. In the evaluation of PD-L1 and PD-1 staining in immune cells, statistical comparisons could not be made and p-values could not be provided due to the limited number of cases in the groups. Therefore, evaluations were made for immune cells by grouping immunohistochemical staining as <1% and ≥1%.

### 2.5. Statistical analysis

Morphological, clinical, and radiological data were incorporated into the obtained data and interpreted with statistical analysis. SPSS 26.0 statistical package program was used for data anaylsis. Descriptive statistics were generated for the evaluation results, including numbers and percentages for categorical variables and mean, standard deviation, minimum and maximum for numerical variables. Kolmogorov–Smirnov or Shapiro–Wilk test was used to assess the conformity of the measurement data to the normal distribution. For measurement variables that fit normally, the t-test in independent groups was utilized. In cases where the measurement variables did not conform to normal distribution, the Mann–Whitney U test or Kruskal–Wallis analysis of variance was applied.. Spearman correlation test was used for the data that did not conform to the normal distribution in the correlation analysis of the measurement data. Chi-square test was used in the analysis of categorical data. In all statistical analyses, the significance levelwas set at p < 0.05.

### 2.6. Ethical approval

The Research Ethics Committee of the Karadeniz Technical University, Faculty of Medicine reviewed and approved all protocols involving human specimens (No. 2020-279).

## 3. Results

**Figure 1:**
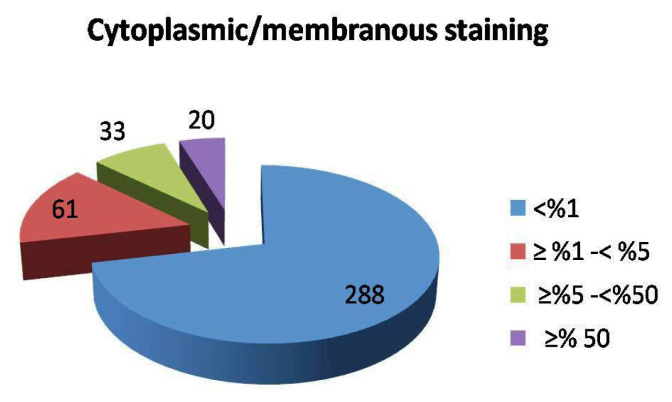
Staining status of tumor cells with PD-L1.

**Figure 2:**
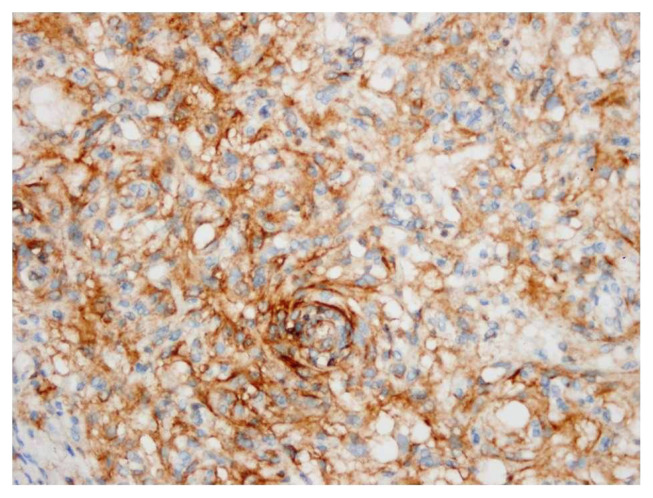
PD-L1 staining at 400× magnification (cytoplasmic/membranous positivity).

Of the 402 patients included in the study, 289 were female and 113 were male. Among these, 271 cases (67.4%) were classified as WHO grade 1; 121 (30.1%) cases as grade 2, and 10 (2.5%) cases as grade 3. Brain invasion was observed in 28 (7.0%) cases. In 336 cases (83.6%), the tumor was located in the supratentorial region; in 43 cases (10.7%), it was in the spinal cord; and in 23 cases (5.7%), it was in the posterior fossa. Hypercellularity was present in 40 cases (10%), small nuclei in 57 (14.2%), macronucleoli in 11 (2.7%), pattern loss in 31 (7.7%), and spontaneous necrosis in 37 (9.2%) cases. Lymphocytes could be easily seen at 100× magnification in 300 of the cases, and in the remaining 102 cases, while in the remaining 102 cases, they were identified upon closer examination at 400× magnification. The status of the cases showing cytoplasmic/membranous staining with PD-L1 is presented in Figure 1. More than 1% cytoplasmic/membranous staining with PD-L1 was observed in 114 cases (Figure 2).

There is a statistically significant difference between staining of PD-L1 in tumor cells and tumor grade (p-value: 0.008). PD-L1 staining was observed in 70/271 (25.8%) grade 1 meningiomas, 37/121 (30.6%) grade 2 meningiomas, and 7/10 (70.0%) grade 3 meningiomas (Table 1). PD-L1 positivity correlates with tumor grade. PD-L1 expression was found to be higher in high-grade tumors.

PD-L1 is positive in 108/391 (27.6%) of the cases without macronucleolus and in 6/11 (54.5%) of the cases with macronucleolus (Table 1). However, although the positivity rate is high in cases with macronucleolus, it is not statistically significant (p-value: 0.051). PD-L1 positivity was compared with cellularity, small nucleus, pattern loss, spontaneous necrosis, brain invasion and recurrence, and no statistically significant difference was found (Table 1). There was no significant correlation between PD-L1 positivity and tumor diameter and age (p-values 0.672 and 0.553, respectively).

PD-L1 was positive in 36.3% (41/72) of men and 23.3% (73/216) of women. Interestingly, PD-L1 was statistically significantly more positive in males than in females (p-value: 0.036).

**Figure 3:**
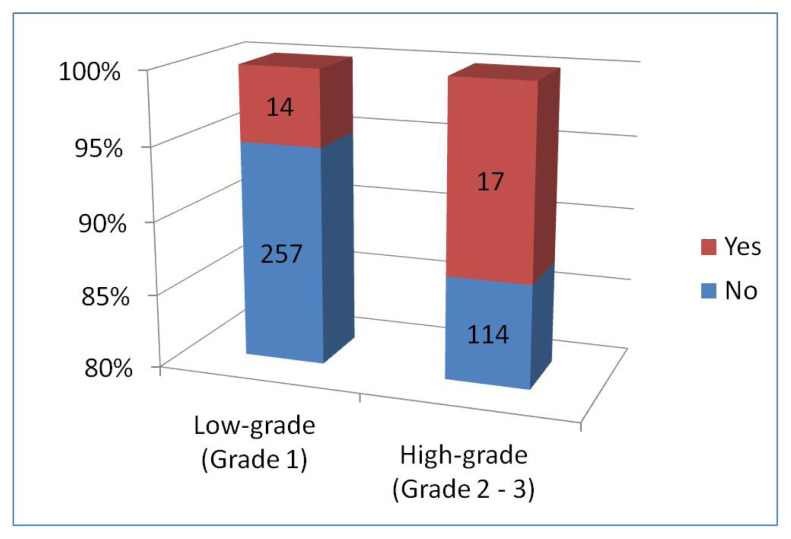
Status of PD-L1 membranous staining of tumor cells in low-grade (grade 1) and high-grade (grade 2 and 3) meningiomas.

Only membranous staining was observed in 31 cases (7.7%). The comparison of PD-L1 and only membranous staining in tumor cells with low-grade (grade 1) and high-grade (grade 2 and 3) meningiomas is presented in Figure 3. It was observed that membranous staining increased statistically in high-grade meningiomas (p-value: 0.006).

The percentage of staining was compared with tumor grade, morphological parameters, and recurrence status in 114 cases with PD-L1 positivity in tumor cells. PD-L1 positivity was more than 50% in 6 out of 12 cases with pattern loss, compared to only 14 out of 102 cases without pattern loss. A statistically significant increase was observed in the percentage of PD-L1 staining in tumor cells in cases with pattern loss (p-value: 0.008). However, in the comparison of the percentage of PD-L1 positivity with tumor grade and morphological parameters, the p-value could not be determined due to the small sample sizes for tumor grade and certain parameters. There was no statistically significant correlation between the percentage of PD-L1 tumor cell positivity and tumor diameter, age, and sex (p-values: 0.857, 0.993, 0.822, respectively).

**Figure 4:**
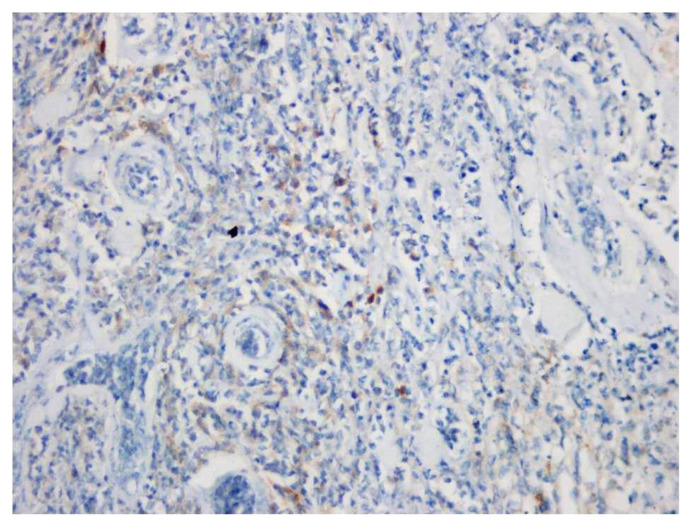
PD-L1 staining at 400× magnification (immune cell positivity).

The number of the cases staining ≥1% with PD-L1 in lymphocytes was 80 (19.9%) (Figure 4), and the number of the cases with <1% or no staining was 322 (80.1%). The comparison of the cases with PD-L1 immune cell positivity with tumor grade, morphological parameters, and recurrence are outlined in Table 2. PD-L1–positive immune cells exhibit a statistically significant increase in cases with spontaneous necrosis (p < 0.001). There was no significant relationship between PD-L1 immune cell positivity and tumor diameter, age, and sex (p-values: 0.590, 0.504, 0.679, respectively).

**Figure 5:**
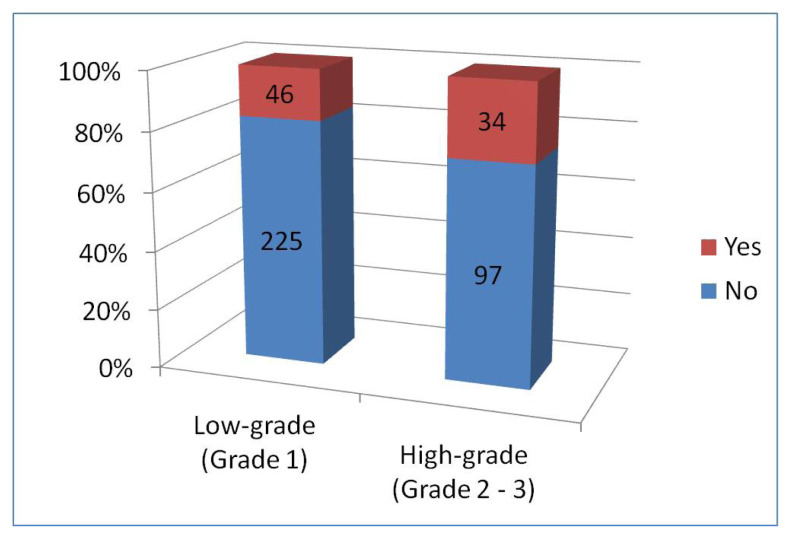
Status of PD-L1 staining of immune cells in low-grade (grade 1) and high-grade (grade 2 and 3) meningiomas.

Immune cell positivity with PD-L1 was 46/271 (17.0%) in grade 1 cases, 30/121 (24.8%) in grade 2 cases, and 4/10 (40.0%) in grade 3 cases. As the tumor grade increases, PD-L1 positivity in immune cells increases. There was no statistically significant difference between PD-L1 immune cell positivity and tumor grade (p-value: 0.055). However, when we grouped meningiomas as low-grade (grade 1) and high-grade (grade 2 and 3) and compared them according to PD-L1 immune cell positivity, statistically significant results were obtained (p-value: 0.045) (Figure 5).

The number of PD-L1–positive immune cells was compared with tumor grade and morphological parameters. In cases with spontaneous necrosis, the number of PD-L1–positive immune cells increased statistically significantly (p < 0.001).

The comparison of PD-L1–positive immune cell count and tumor grade is outlined in Table 3. Statistically significant difference was found between tumor grade and PD-L1–positive immune cell count (p-value: 0.045).

**Figure 6:**
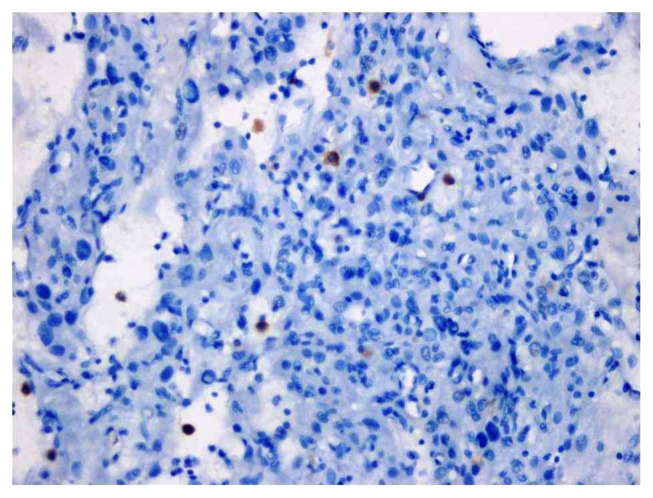
PD-1 staining at 400× magnification (immune cell positivity).

Staining with PD-1 was observed in at least 1 and at most 30 immune cells in meningiomas (Figure 6). The number of the cases showing ≥1% immune cell staining with PD-1 was 36 (9.0%). In 366 cases (91.0%), no staining was observed in immune cells with PD-1. The comparison of PD-1 immune cell staining with tumor grade, morphological parameters and recurrence is presented in Table 4. PD-1 positivity increases in cases with brain invasion (p-value: 0.017). There was no statistically significant result between PD-1 immune cell positivity and tumor grade (p-value: 0.854). No statistically significant results were obtained between PD-1 immune cell positivity and tumor grade (even when we grouped meningiomas as low-grade (grade 1) and high-grade (grade 2 and 3) (p-value: 0.636). No statistically significant correlation was found between the cases showing PD-1 immune cell positivity and tumor diameter, age, and sex.

The comparison of the number of PD-1–positive immune cells with tumor grade morphological parameters and recurrence in all cases is outlined in Table 5. There is a correlation between PD-1–positive lymphocyte count and brain invasion (p-value: 0.018). The number of PD-1–positive immune cells was compared with tumor grade, but no statistically significant results were obtained (p-value: 0.897). Furthermore, there was no statistically significant correlation between the number of PD-1–positive immune cells and tumor diameter, age, and sex (p value 0.107 – 0.093 – 0.959, respectively).

## 4. Discussion

Meningiomas are the most common adult primary tumors of the central nervous system and have a relatively high recurrence rate. About 12% of grade 1 meningiomas recur within 5 years after total resection, while 19% recur in 10 years [13]. Even after total resection, the overall recurrence rate for grade 2 tumors in all localizations is 29%–40% within 5 years [13]. Although recurrence is common, managing patients with recurrent tumors or anaplastic meningioma, particularly those who are inoperable and have previously undergone radiotherapy, has proven challenging due to the lack of effective chemotherapy and other medical treatment methods. Meningiomas arise from the meninges located outside the blood-brain barrier and therefore can be directly targeted by antibody-mediated immunotherapy. In the literature, studies on immunocheckpoint molecules in meningiomas are very limited and there are some conflicting results.

In the study by Du et al., PD-L1 was found to be positive in 40% of grade 1 meningiomas, 60% of grade 2 meningiomas, and 77%–88% of grade 3 meningiomas. It was reported that PD-L1 expression increased in grade 2 and 3 meningiomas. However, this study did not mention whether the immunohistochemical staining was membranous or cytoplasmic [14]. In the study by Everson et al., although PD-L1 was 25% positive in grade 3 meningiomas, no staining was observed in grade 1 and 2 meningiomas [15]. These differences in PD-L1 expression may stem from the specific cell populations analyzed. The presence of PD-L1–positive macrophages within the tumor, alongside PD-L1 expression in tumor cells, might be the reason for the divergent PD-L1 results observed among patients [16]. In Johnson’s study, PD-L1 was positive in only 1 (3%) of 31 grade 1 cases, only 1 (6%) of 16 grade 2 cases, and 2 (18%) of 11 grade 3 cases [13]. In the study by Han et al., similar to Johnson’s study, it was concluded that PD-L1 is expressed in meningiomas and that its expression increases with the degree of meningiomas [16]. Han et al. also stated that high expressions of PD-1 and PD-L1 were associated with worse survival in high-grade meningiomas, independent of tumor grade, resection rate, and previous recurrence [16]. In our study, PD-L1 expression was found in 38.4% of meningiomas at varying rates, more than 1%. PD-L1 expression was observed in 25.8% of grade 1 meningiomas, 30.6% of grade 2 meningiomas, and 70.0% of grade 3 meningiomas. It has been observed that PD-L1 expression increases as the grade increases. In our study, PD-L1 expression was evaluated by membranous staining in tumor cells as in some other tumors (lung, breast). The membranous expression of PD-L1 was statistically significantly increased in high-grade (grade 2 and 3) meningiomas. In addition, interestingly, PD-L1 in our study shows statistically significantly more positivity in males than in females.

Yunnica et al. stated that strong VEGF-A and PD-L1 expressions are associated with the presence of high-grade meningioma and that VEGF-A and PD-L1 expressions can be regarded as factors affecting the aggressiveness of meningioma [17]. In our study, PD-L1 expression increased with tumor grade. In the study of Karimi et al., PD-L1 expression was observed in 27% (11/41) of grade 1 cases, 47% (23/43) of grade 2 cases, and 67% (6/9) of grade 3 cases [18]. In this study, they reported that PD-L1 is a marker that can predict tumor recurrence in meningiomas [18]. In our study, however, no statistically significant relationship was found between PD-L1 expression and recurrence. In other words, PD-L1 antibody cannot predict recurrence.

Cytoplasmic/membranous staining was evaluated in studies with PD-L1. In our study, membranous staining alone was also evaluated. Only membranous staining was found to be statistically significantly increased in high-grade meningiomas. In other words, only membranous PD-L1 expression also indicates high-grade meningiomas and indicates tumor aggressiveness. Similarly, Bi et al. found that PD-L1 expression was associated with negative prognosis in high-grade meningiomas [3].

In our study, the relationship of morphological parameters with PD-L1 was also examined and no statistically significant results were observed. However, it was observed that the percentage of staining increased statistically significantly in cases with pattern loss.

In the study by Karimi et al., PD-L1 expression in meningiomas was observed in 43% of both tumors and immune cells [18]. In our study, immune cell positivity with PD-L1 was observed in 80 cases (19.90%). In addition, it was observed that PD-L1 positivity in immune cells increased as the tumor grade increased. However, a statistically significant p-value could not be obtained. On the other hand, PD-L1 immune cell positivity increases statistically significantly in high-grade (grade 2 and 3) meningiomas. A statistically significant increase in PD-L1 expression was also observed in cases with spontaneous necrosis.

In the study by Everson et al., PD-1 expression was observed in lymphocytes in 24/52 (46%) cases of meningiomas. PD-1 showed similar expression in different grades of meningiomas [15]. In our study, expression of PD-1 in lymphocytes was observed in 36/402 (9%) cases. In our study, similar to the study by Everson et al., the expression of PD-1 in meningiomas did not show a significant difference according to tumor grade. On the other hand, in our study, a significant relationship was observed between PD-1 immune cell positivity and brain invasion. There is a significant increase in the number of PD-1–positive lymphocytes in cases with brain invasion.

In the study by Du et al., it was observed that the number of lymphocytes expressing PD-1 decreased in high-grade meningiomas [14]. In our study, although it was not statistically significant, the number of PD-1 positive lymphocytes decreased as the tumor grade increased, on the other hand, PD-L1 expression showed a statistically significant increase in high-grade tumors.

Meningiomas are “benign” tumors that are typically treated with surgery and radiation. However, treatment options are very limited in relapsed or unresectable cases because they are resistant to chemotherapy. Recent advances in the treatment of cancers with immunotherapy have focused on immune checkpoint blockade. There are recent studies supporting the use of immunotherapy as a potentially effective treatment strategy for meningiomas.

Studies show that PD-L1 level in tumor cells can be a guide in the treatment (immunotherapy) plan. Depending on the type of cancer, patients with PD-L1–positive tumors respond 2–3 times higher to immune checkpoint inhibitors than negative ones [19].

Treatment of meningiomas with a monoclonal antibody targeting PD-L1 demonstrated a significant reduction in tumor size in a recently published case report by Gelerstein [20]. A tissue diagnosis was not made in this case report. Response to treatment was monitored by radiologic imaging. Similarly, in a case published by Dunn et al., it was shown that there was a significant reduction in tumor size and edema after treatment with a monoclonal antibody targeting PD-L1 [21]. Dunn’s case was recurrent atypical meningioma. In the study by Giles et al., it was reported that PD-L1 may be the treatment target in grade 3 meningiomas [22]. In the study by Li et al., it was reported that the anti-PD-L1/PD-1 combination may be the target of treatment in high-grade meningiomas [23].

Karimi et al. stated that immunohistochemical PD-L1 expression was irregular and showed inter- and intratumoral heterogeneity. Additionally, they stated that the decrease in immune cells, high PD-L1 expression, and high tumor mutation burden found in high-grade meningiomas indicate an immunosuppressive tumor microenvironment. They concluded that these data provide a rationale reason for various ongoing trials investigating checkpoint inhibitor therapy in high-grade and refractory meningiomas [24]. In their study, Filippone et al. stated that PD-L1 may play an important biological role in the treatment of grade 2/3 meningiomas in which radiotherapy fail [25].

Genetic changes are thought to be extremely important in the treatment of meningiomas. Although meningiomas do not have a high mutation load, there is a subgroup with higher somatic mutation rates that may be better candidates for immunotherapy [26,27]. For example, meningiomas commonly have isolated monosomy 22/del(22q) mutations that increase in tumor-infiltrating M1-subtype macrophages, NK cells, and T cells [28,29]. Recently, PD-L1 levels were found to be significantly higher in tumors with TRAF7 mutations than in tumors without TRAF7 alterations. This result suggests that patients with TRAF7 mutations may benefit from immunotherapy [30].

## 5. Conclusion

In our study, PD-L1 expression correlates with tumor grade. Only membranous staining was seen in 7.7% of the cases. The cases showing staining with PD-L1 in lymphocytes was 19.9%. In addition, PD-L1 positivity also increased in immune cells in high-grade meningiomas and spontaneous-necrosis-positive cases. Staining with PD-1 in immune cells was ≥1% in 9.0% of the cases. PD-1 immune cell expression was correlated with brain invasion.

These results show that immune checkpoint molecules with varying levels of expression can be used as a prognostic and predictive biomarker, as well as an important target for therapy. As a multitargeted approach utilizing various components of the immune microenvironment, combined immunotherapeutic agents can lead to favorable outcomes in cases of high-grade, frequently recurring, and unresectable meningioma.

In conclusion, gaining insights into the status of immune checkpoint molecules, understanding the genomic characterization of tumors, accurate grading, careful patient selection for appropriate treatment, and increasing clinical experience with immune checkpoint blocking agents could pave the way for discovering effective treatments for patients with aggressive meningiomas.
